# Editorial: Precision/Personalized Pediatric Oncology and Immune Therapies: Rather Customize Than Randomize

**DOI:** 10.3389/fonc.2020.00377

**Published:** 2020-03-24

**Authors:** Michal Kýr, Giannoula Lakka Klement, Lenka Zdrazilova-Dubska, Regina Demlova, Dalibor Valik, Ondrej Slaby, Irene Slavc, Jaroslav Sterba

**Affiliations:** ^1^Department of Pediatric Oncology, University Hospital Brno, Brno, Czechia; ^2^Faculty of Medicine, Masaryk University, Brno, Czechia; ^3^CSTS Health Care, Toronto, ON, Canada; ^4^Floating Hospital for Children at Tufts Medical Center, Boston, MA, United States; ^5^Department of Laboratory Medicine, Masaryk Memorial Cancer Institute, Brno, Czechia; ^6^Department of Pharmacology, Faculty of Medicine, Masaryk University, Brno, Czechia; ^7^Department of Clinical Trials, Masaryk Memorial Cancer Institute, Brno, Czechia; ^8^Department of Pathology, University Hospital Brno, Brno, Czechia; ^9^Central European Institute of Technology (CEITEC), Masaryk University, Brno, Czechia; ^10^Department of Pediatrics and Adolescent Medicine, Medical University of Vienna, Vienna, Austria

**Keywords:** personalized medicine, precision oncology, pediatric oncology, trial models, drug models, personalized precision pediatric oncology, clinical trial designs

Personalization of treatment based on biological markers is being utilized in clinical medicine with increasing frequency. This trend, despite an effort to identify possible common patterns, reflects the reality that no two patients are alike, and no single clinical course is identical; not even within a group of seemingly similar patients (1). There are numerous clinical variations related to host or environment-dependent factors. Numerous examples of these interpersonal differences have been recognized with drugs such as pain-control medications, heart medications, or antimicrobials. The differences have been attributed to increased pharmacometabolic capacity, to different individual microbiomes and to genetic differences between individuals (2). The latter has led to development of an entirely new specialty—pharmacogenomics. While this clinical heterogeneity is well-appreciated in most major medical specialties, clinical oncology seems to represent, surprisingly enough, one of the exceptions (3, 4).

Individualized treatments aim to optimize patient outcomes based on specific knowledge about diseases and their biological heterogeneity (5). This individualization of therapy is being adopted even in adult oncology where, at least traditionally, new therapeutic directions depended on success in large randomized clinical trials. Even in cancers where the numbers of adult patients are sufficient for large randomized double-blind clinical trials, the recent trends are to select the most suitable, genetically homogeneous, target population. This trend has been more inherent to pediatrics, where malignancies are implicitly considered rare diseases. However, the smaller populations and a personalized approach, has led to a very small number of drugs being approved for pediatric indications. The small number of patients and more personalized combinations of drugs tended to complicate statistical analysis and created problems for providing evidence of treatment efficacy in children with rare malignancies (Kyr et al.).

When a large homogeneous population can be used—a randomized, double blind, placebo controlled trial should remain the gold standard. However, this is rarely possible considering cancer heterogeneity and interpersonal differences in drug response. In pediatrics, the numbers of patients are relatively small and the diseases heterogeneous. The process of randomization and blinding were originally developed to protect the subjects and the investigators from pre-existing subjective preferences for a procedure or a compound under evaluation (6, 7). Randomization was intended to minimalize the effect of confounders, to achieve comparable groups and to permit calculation of an unbiased estimate of the treatment effect. While the use of “blinding” in order to eliminate bias is obvious, there is another important tool that makes randomized trials powerful with regard to rendering reliable and unbiased results. It is the balancing effect between investigated groups, especially with respect unknown covariates that cannot be easily eliminated through model adjustments nor stratification. As stressed above, randomization requires sufficient number of patients and adequate sample size to work. A test sample >200 is said to be less likely to be imbalanced for an important covariate (8). But in rare diseases, where the sample sizes are small (rarely more than a hundred), the usefulness of randomization for balancing of the groups is lost. Similarly, randomized, double blind, placebo-controlled trials may not be suitable for populations that are selected on a common, but infrequent genetic alteration(s). Those groups are also quite small. While the gold standard of clinical trials, a randomized, double blind, placebo controlled trial, may have made logical sense in the era before genomics, it may need to be modified for the era informed by testing for individualized traits and smaller groups.

The concept of time-dependent variations is equally important (9). As documented in numerous recent publications, variations within an individual and implicitly, within the individual's *macroscopic tumor*, occur at velocity rates that cannot be measured by any contemporary techniques (10). It is this variability that constitutes a fundamental concern with the use of treatment group randomization. For a set of individuals being randomized using current rules, a critical prior assumption is made that *all randomized individuals are, and will remain, biologically homogenous*, and any further events can only be related to the time point of randomization. A further assumption is then made that *no change within the set of investigated subjects occurs during the study period* except the changes due to treatment. This is not true for cancers, which are known to evolve through continuously accumulating additional genomic alterations through mutations. Consequently, even if randomization was performed at baseline, the randomization effect is lost in any repeated evaluation during subsequent phases of such trials (11).

Single patient trial designs or “*N* = 1” trials (12) are an alternative to population-based clinical trials, but a broad clinical application of this approach is hindered by the absence of a standardized work-up. Current practices are based on physician-specific or institution-enabled assessments of the biological characteristics of the patient and of the cancer tissue. This usually occurs in the form of a multidisciplinary institutional expert consensus referred to as “tumor boards” (13). This personalized treatment approach allows for consideration of disease heterogeneity as well as of time-dependent variations. This *clinical plasticity*, allows for treatment to be modified at various phases of the patient's journey based on disease course or on the patient's pharmacometabolic capacity to tolerate the selected treatment. The much-needed standardization of the pre-treatment workup of a patient selected to undergo personalized therapy would enable collection of outcomes from these “*N* = 1” trials in pediatric cancer across many institutions, enable statistical analysis, and provide evidence for changing therapeutic paradigms.

Another issue arising in rare diseases, and therefore personalized pediatric oncology, is the identification of *future target population likely to benefit from a trial result*—the so called “patient horizon” (14). Patient horizon is either the number of patients in the trial, or the number who have the condition under treatment. This well-known concept is rarely utilized. To improve understanding of this concept let us take an extreme situation where all patients from the target population were randomized in 1:1 ratio for effective and ineffective treatment. In this case half of the patients are forced to receive an ineffective treatment as a price for knowing the absolute truth about the relative treatment efficacy between the two treatments. Yet, the same result could be obtained by giving either of the treatment randomly without any knowledge.

An optimal size of a trial balances both extremes and maximizes the number of patients who benefit. The exact number of patients may not be known, but the order of magnitude of the optimal number can be calculated using *the square root of the patient horizon size* for a simple trial design. For example, for a finite population of 1,000 subjects, the optimal size of a trial is a few tenths. Considering disease rarity, especially in the era of molecular medicine, the issue of the target population size (the patient horizon) becomes relevant not in pediatric oncology, but in medicine in general (14).

## Issues to Be Addressed

There are two principal issues to be addressed in current cancer medicine pertaining to:

- Regulatory mechanisms of drug approval and market authorization.
- Evaluation of real-life clinical efficacy.

### Regulatory Considerations

A newly proposed drug approval marketing authorization pathway shall require an initial “Candidate Medicinal Product Safety Evaluation” (CMPSE, currently Phase I) and subsequent “Dose Defining Study” (DDS, currently Phase II). As we explained above a current medicinal product approval pathway is mechanistically “drug-centric” as the present practice relies on the ability of a Phase III clinical trial to provide evidence that the addition of a single compound to a standard treatment regimen is of clinical benefit *leading to marketing authorization*. This approach has become so biased that most resulting Phase III registration trial data do not provide clinically meaningful benefit (3); *on top of that, testing for “me-too” drugs toward endpoints as “substantial equivalence or non-inferiority” is vastly contributing*. Furthermore, it disregards the clinical need for different pathways for approval of medicines intended for use (A) in the entire world population (e.g., vaccines, antipyretics, pain killers, etc.) and for those intended for (B) specific subpopulations (e.g., LDL-C marker based treatments). In the latter setting, clinical laboratory diagnostics (CDx) are used as a guide or companion to a medicinal product to determine its applicability to a subject. A regulatory approval of medications targeting (C) somatic mutations, and/or (D) diseases that follow Mendelian inheritance or germline mutation (e.g., tyrosinemia type I. and the drug nitisinone) requires a special approval pathway and expedited translation to clinical practice. Summing up from a regulatory point of view, the testing phases CMPSE Phase I + DDS Phase II would allow for *conditional medicinal product (pre)approval*.

Real-life clinical practice-based evaluation should then focus on designing “patient-centric” treatment strategies. Considering there are about 300 active drugs in oncology, and the number of 2 drugs combinations is about 45,000, or 4.4 million combinations for 3 drug combinations, Phase I testing for all these drug combinations is neither feasible nor realistic. New models such as: (i) identification of smaller pediatric cancer patient cohorts likely to benefit from a specific treatment because they have the relevant gene alteration(s), or (ii) the increasing use of multilayer profiling (markers) to diagnose, classify and monitor response in pediatric cancers (Fedorova et al.; Polaskova et al.) are therefore gaining in popularity. There is a need to validate combination treatment strategies, not just individual drugs or individual biomarkers. Attention should be directed at studying drug dosing in respective preclinical models and at identifying optimal biological dose rather than persist with the present maximum-tolerated dose. With most targeted agents, a target occupancy dose, i.e., dose required to stop/minimize pathway phosphorylation and RP2D /dose used in clinical setting (15) is the more appropriate identifier of a clinically relevant dose. As noted in many pre-clinical studies, combinations of targeted agents are often synergistic, and potentiate the effects of chemotherapy. A very good example of how combination therapy dosing can negatively influence the overall success of an innovative drug is the Mylotarg (gemtuzumab ozogamicin, alias GO) story. *Gemtuzumab ozogamicin is a recombinant humanized IgG4 kappa antibody that is used to treat CD33 positive AML. It is conjugated with calicheamicin derivative, a cytotoxic antitumor antibiotic. The drug was initially tested in a randomized controlled trial leading to FDA approval via accelerated review in May 2000. However, the drug had intolerable toxicity and mortality at the 9 mg/m*^2^ *dose, and was voluntarily withdrawn from the market on 15th October 2010. It was subsequently tested at a much lower drug dose (3 mg/m*^2^ *instead of 9 mg/m*^2^*) and was shown to be just as effective with greatly improved safety profile. GO was therefore re-approved by the FDA on 1st September 2017 at lower dose* (16). Taken together, if a new compound allowed to enter the real-life clinical practice-based evaluation (i.e., CMPSE + DDS passed) brings clinically meaningful benefit, this will lead to the full marketing authorization and consequently, reimbursement of such a novel compound.

The use of chemotherapy in combination with a targeted biological agent is a commonly employed approach for enhancing the ability of chemotherapy to fight cancer. Commonly, the assumption that the inhibitory effect of the biological agent would be additive to the effect achieved by traditional chemotherapy or radiation is made. However, because of the synergistic action, the addition of a targeted biologic agent to a maximum tolerated dose (MTD) of chemotherapy, may make an already maximally toxic regimen almost lethal. In most cases, any benefit of tumor response ends up being concealed by unacceptable toxicities, and no overall survival benefit is seen. Yet, because the present design of clinical trials permits modification of only one variable between the two study arms, the dose of chemotherapy in the experimental treatment arm is rarely modified. The use of metronomic chemotherapy, with its goal of long-term “tumor control,” lower toxicity, and prevention of tumor progression (rather than immediate reduction in tumor size), may represent a more realistic strategy for testing targeted and immune therapies as add on to chemotherapy (13). However, because this low toxicity regimen can have a delayed onset of radiologically visible effect, it is often abandoned too early for a patient to benefit. An example of how biomarker assessment can help document the effects of targeted therapy earlier than it could be documented radiologically is provided in this issue (Polaskova et al.) discussing three patients with multiply relapsed Burkitt lymphoma treated with personalized therapy and their response being monitored using target phosphorylation.

In summary, data for real-life evidence-based medicine addressing patient-focused clinical efficacy can be derived from time-dependent single-case designs. The new comprehensive efficacy evaluation model we present here, should be focused on treatment strategies using drug combinations rather than testing a single-compound within a randomized setting. We should modify the Phase III wherever feasible. The drug approval pathway should consist of “Candidate Medicinal Product Safety Evaluation” (previously Phase I) and “Dose Defining Study” (previously Phase II). This will bypass the often futile end-of-life enrollments in single drug clinical trials and bring about substantial cost reductions in development and implementation of new medicinal anticancer compounds to the market.

## Author Contributions

All authors listed have made a substantial, direct and intellectual contribution to the work, and approved it for publication.

### Conflict of Interest

The authors declare that the research was conducted in the absence of any commercial or financial relationships that could be construed as a potential conflict of interest.
